# Anabolic Steroid-induced Mania

**DOI:** 10.7759/cureus.3163

**Published:** 2018-08-20

**Authors:** Daniel G Franey, Eduardo D Espiridion

**Affiliations:** 1 Psychiatry Clerkship, Frederick Medical Center/West Virginia University, Reading, USA; 2 Psychiatry, West Virginia School of Osteopathic Medicine, Martinsburg, USA

**Keywords:** anabolic steroids, mania, cannabis

## Abstract

There are numerous reports of the psychiatric effects of anabolic-androgenic steroid (AAS) use. However, these effects have not been clearly elicited in controlled clinical trials. This discrepancy is largely due to the presence of a variety of synergistic factors seen in the real-life setting of AAS abuse.

In this case, we report a patient in acute mania admitted to Frederick Memorial Hospital in Frederick, Maryland. He had no prior history or family history of manic episodes. His symptoms were refractory to initial pharmacologic intervention. The onset of his symptoms was likely related to the initiation of AAS use. However, his symptoms were likely potentiated by heavy daily cannabis use. The patient showed a gradual improvement over the second week of his hospitalization. He was discharged on antipsychotics and scheduled to follow up with a therapist and psychiatrist.

## Introduction

Anabolic-androgenic steroids (AAS) are compounds that act on testosterone receptors that have been used to increase muscle size and strength with the goal of improving athletic performance or enhancing physical appearance. Therapeutically, they are used to treat human immunodeficiency virus (HIV)-related muscle wasting disease, severe burn injuries, and constitutional growth retardation among other things [1]. To the public, AAS use is most familiar in the setting of professional athletes penalized for testing positive in a performance-enhancing drug screening; however, there is a large population outside of professional athletics involved in AAS use. A 2006 survey, which looked to identify trends in the habits of AAS users, found that nearly 80% of AAS users are non-athletes who use these drugs for cosmetic reasons [2]. While the physical side effects are well documented, the psychiatric side effects and the circumstances that precipitate them are still frequently studied. We report a patient in acute mania likely due to the initiation of AAS use that is potentiated by heavy daily cannabis use.

## Case presentation

The police brought a 30-year-old man to the emergency department at Frederick Memorial Hospital in Frederick, Maryland, at the request of his mother. She reported that he had been unusually volatile for the past three weeks with multiple violent outbursts. In addition, there were episodes where she witnessed the patient shouting at the sky. She made the decision to call emergency services in response to her son’s homicidal threats toward his ex-girlfriend. According to her, the patient had no history of manic episodes or any family history of mood disorder. She said his current symptoms began when he started attending a new gym three weeks prior to his hospital admission. She suspected that he had begun injecting anabolic steroids.

In the emergency department, the patient’s complete blood count and basic metabolic panel were within normal limits. His urine toxicology screen tested positive for cannabis. The patient’s past medical history was significant for anxiety, depression, post-traumatic stress disorder (PTSD), opioid addiction with methadone maintenance therapy, and hepatitis C. His time in the emergency department was marked with extreme agitation that culminated in attempts to fight with the staff. He was treated with haloperidol 5 mg IM and lorazepam 2 mg IM. He was admitted to the behavioral health unit.

The patient was initially started on olanzapine 10 mg PO QD. The patient was unable to give a history for the first week of his hospitalization. On examination, he presented with expansive mood, pressured speech, psychomotor agitation, racing thoughts, inflated self-esteem, and decreased need for sleep. He was often seen pacing the hallways while talking loudly to himself and singing. His actions toward female staff were inappropriate and signaled that he was not cognizant of personal boundaries. He made verbal threats to staff and other patients and was not verbally redirectable. On two occasions where the patient became violent, ziprasidone 20 mg IM and lorazepam 2 mg IM were needed. He showed little improvement in his symptoms going into the second week of his hospitalization. His olanzapine was titrated to 15 mg QD, and chlorpromazine 100 mg PO QID was added.

At each attempt to ascertain the patient history, a similar pattern was observed. The patient quickly went off on tangents relating to marijuana and bodybuilding, which seemed to be a source of pride for him. He said he has used cannabis for recreation and for the treatment of PTSD from time spent in prison. He is the owner of a medical marijuana card and has used the substance multiple times a day since he was a teen in a variety of methods, including edibles, vaporization, smoking, and ingestible oils. He also grows and sells marijuana and claims he has a reputation for being the best. His agitation level rose greatly when he spoke of people who doubted his abilities. He would then tense up and begin to speak of his training in martial arts and all the time he had spent building muscle in the gym. When asked directly about anabolic steroid use, he would change the subject, deny it altogether, or say he only took supplements. Staff reported, however, that the patient had spoken of “Deca” (a colloquial name for Deca Durabolin, an anabolic-androgenic steroid) multiple times in his pressured, tangential conversations with them. The patient’s mother had since spoken with friends of the patient who said he had been injecting anabolic steroids once weekly at an unknown dose. Although the mother requested a laboratory test for anabolic steroids, it was not available at the hospital. She had contacted poison control about the availability of the test and planned to pursue it further when the patient moved to the outpatient setting.

The patient’s mania and psychosis improved gradually in the final week. He was discharged on chlorpromazine 100 mg PO TID and olanzapine 15 mg PO QD and scheduled with follow-up appointments with a psychiatrist and a therapist. He was not readmitted to any psychiatric unit since he was discharged.

## Discussion

The documented psychiatric effects of AAS use include aggression, cognitive impairment, mania, depression, and psychosis [3]. These symptoms can occur within days of initiating AAS use [4]. In addition to the acute effects, the long-term effects of AAS use have been a focus of recent studies. A retrospective study of Swedish elite athletes that examined the long-term use of AAS showed that those with a history of AAS use were more likely to have sought professional expertise with mental health problems, especially anxiety and depression [5]. Furthermore, dependence and withdrawal symptoms can arise with long-term steroid use [6]. Thus, it is evident that the psychiatric effects of anabolic steroid use are expansive and subject to many variables. Studies are often unable to elicit the complete spectrum of effects seen in real-life AAS use due to methodology limits, such as controlled doses of a single anabolic steroid and participant exclusionary criteria, including previous drug abuse and psychiatric illness [7].

In particular, controlled doses are limiting criteria because stacking, which refers to the practice of administering a dose, which is often made up of multiple different anabolic steroids that can be 10-100 times larger than the therapeutic dose, is a common practice with AAS abuse [8]. Therefore, case reports are useful in observing the real-life spectrum of the effects related to AAS use. In a case report by Papazisis et al., a 25-year-old male body builder was hospitalized for a manic episode with psychotic features [9]. He had a history of depression but no history of drug abuse. Mania and psychosis occurred shortly after increasing his AAS dose to > 2 g daily. For comparison, the healthy male produces 2.5-11 mg of testosterone daily, and the therapeutic dose for treatment for muscle wasting in HIV is approximately 100-150 mg daily [10-11]. Unfortunately, the subject of the case later committed suicide as a result of AAS discontinuation and subsequent withdrawal-related depression.

With the above case report and other laboratory studies, it appears that excessive dosing is a leading factor in the induction of manic symptoms [3]. However, there have been reports of manic symptoms even within the therapeutic dosing of AAS. For example, a 28-year-old man with acquired immunodeficiency syndrome (AIDS) was admitted with manic symptoms after beginning the prescribed testosterone patch therapy (2-6 mg daily) one month earlier [12]. This patient had a history of bipolar II disorder, which suggests a lower threshold to AAS-induced mania in certain populations.

Randomized controlled trials that aimed to capture AAS effects at doses truer to real-world doses did not note overwhelming evidence of mania or psychosis. In 2000, Pope et al. performed a randomized controlled trial of supraphysiologic doses of testosterone in 56 normal men aged 20-50 that showed only 4% of participants became hypomanic after the completion of a crossover study in which AAS doses were increased from 150 mg weekly to 600 mg weekly over six weeks [13]. Bhasin et al. examined the physical effects of a 600 mg weekly dose of testosterone over 10 weeks [14]. Compared to the placebo groups, no significant changes in mood or behavior were seen. Notably, these studies excluded participants with a history of drug abuse or a history of psychiatric illness.

The heavy daily cannabis use with our patient is particularly concerning due to the reports of cannabis-induced mania. In 2015, Gibbs et al. performed a meta-analysis of cannabis use and mania symptoms, which reviewed the results of six articles [15]. Their conclusions suggest that cannabis use may contribute to the development of mania over time and may worsen the occurrence of manic symptoms in bipolar disorder. This finding is illustrated in a case where a 17-year-old male with no psychiatric history develops manic symptoms and is admitted to the hospital after greatly increasing his marijuana intake [16]. Like our patient, this patient failed initial medication trials. His symptoms did not improve until week three of hospitalization.

In a naturalistic study of 33 participants by Pope et al., manic symptoms were observed in nine participants and psychotic symptoms were observed in five participants [17]. This study allowed for dose stacking and did not exclude participants based on an underlying psychiatric condition or a history of substance abuse. In those participants that experienced mania or psychosis, stacking was a common denominator. Unfortunately, the amount of Deca Durabolin used by our patient could not be determined. While our patient’s suspected weekly injection seems far from the excessive daily doses seen in some case reports, the role of stacking, in this case, cannot be completely determined. However, the patient’s heavy cannabis use should not be ignored as a factor in his mania induction.

The proposed mechanism for the effects of AAS on mood is via the modulation of the metabolism of tryptophan and serotonin in the brain, which leads to symptoms of euphoria, arousal, and decreased anxiety at low doses, and leads to symptoms of aggression, anxiety, depression, mania, and psychosis at high doses [18]. Additionally, chronic cannabis use leads to increased levels of pregnenolone metabolites that are thought to contribute to psychiatric illness and leads to an increased excitatory state in the brain [19]. Furthermore, the irregularities in neurosteroid homeostasis were only seen in cannabis use disorder and, to a lesser extent, opioid use disorder. The combination of the actions of these neuroactive substances could have had a synergistic effect that precipitated our patient’s manic episode.

## Conclusions

With no known history of mania, the patient met the criteria for a manic episode. Based on the history, the probable inciting factor was the initiation of AAS injections. Given the patient’s history of substance abuse—particularly heavy cannabis use, it is likely the patient had an increased susceptibility to the psychiatric effects of AAS use.
